# A Comprehensive Genome-Wide Study on Tissue-Specific and Abiotic Stress-Specific miRNAs in *Triticum aestivum*

**DOI:** 10.1371/journal.pone.0095800

**Published:** 2014-04-23

**Authors:** Ritu Pandey, Gopal Joshi, Ankur R. Bhardwaj, Manu Agarwal, Surekha Katiyar-Agarwal

**Affiliations:** 1 Department of Plant Molecular Biology, University of Delhi South Campus, New Delhi, India; 2 Department of Botany, University of Delhi, Delhi, India; Texas Tech University, United States of America

## Abstract

Productivity of wheat crop is largely dependent on its growth and development that, in turn, is mainly regulated by environmental conditions, including abiotic stress factors. miRNAs are key regulators of gene expression networks involved in diverse aspects of development and stress responses in plants. Using high-throughput sequencing of eight small RNA libraries prepared from diverse abiotic stresses and tissues, we identified 47 known miRNAs belonging to 20 families, 49 true novel and 1030 candidate novel miRNAs. Digital gene expression analysis revealed that 257 miRNAs exhibited tissue-specific expression and 74 were associated with abiotic stresses. Putative target genes were predicted for miRNAs identified in this study and their grouping into functional categories indicated that the putative targets were involved in diverse biological processes. RLM-RACE of predicted targets of three known miRNAs (miR156, miR160 and miR164) confirmed their mRNA cleavage, thus indicating their regulation at post-transcriptional level by the corresponding miRNAs. Mapping of the sequenced data onto the wheat progenitors and closely related monocots revealed a large number of evolutionary conserved miRNAs. Additional expression profiling of some of these miRNAs in other abiotic stresses underline their involvement in multiple stresses. Our findings provide valuable resource for an improved understanding of the role of miRNAs in stress tolerance as well as plant development.

## Introduction

Bread wheat (*Triticum aestivum L*, AABBDD, 2n = 6x = 42) is a vital food crop throughout the world. However, there is an enormous gap between the supply and demand of wheat grains across the globe, especially in the Asian region [1]. This gap is mainly attributed to the constraints imposed by both pre-harvest and post-harvest determinants. The wheat crop is challenged by several biotic and abiotic factors during its life cycle. These detrimental effects are exacerbated when plants are exposed to a combination of multiple, simultaneous or sequential, stress factors [2]. To devise novel effective molecular strategies for enhancing stress tolerance, understanding the mechanism of stress perception and downstream gene regulatory pathways is of paramount importance. Several investigations have identified the molecular components that act, either individually or in liaison with other molecules, to impart stress adaptation in wheat [3], [4]. These components are, in turn, themselves regulated and therefore identification of such regulators, which fine tune the expression levels of stress-associated components and subsequently stress signaling pathways, would provide insights into the molecular mechanism of stress tolerance in wheat.

MicroRNAs (miRNAs) are crucial ubiquitous regulators of gene expression at transcriptional, post-transcriptional and translational level [5]–[7]. Functionally, miRNAs are associated with diverse biological roles, from regulating development to assisting plants combat harsh environmental conditions [8]. Contributions made by many research groups have generated a wealth of information on structure, function and regulation of miRNAs in model plants such as *Arabidopsis*, rice, *Medicago* and *Brachypodium*. Though identification of known miRNAs is relatively easier, identification of novel miRNAs has remained a challenge in the absence of complete reference genomic sequences of many economically important plants, including wheat. Nevertheless, due to the sequence conservation of miRNAs across large evolutionary distances, computational methods have been successful in identifying few miRNAs in wheat [9]–[15]. The miRBase registry (v20; http://www.mirbase.org/cgi-bin/browse.pl) contains 7385 (4025 unique) mature miRNA sequences from 72 plant species, with only 42 representative miRNA entries for *Triticum aestivum*. With the availability of next generation sequencing (NGS) there has been an unprecedented surge in genomic resources, which has subsequently led to the discovery of novel miRNAs in several non-model plant species. Few recent studies involving high-throughput sequencing have revealed additional miRNA sequences in wheat and closely related species. Wei et al. [16] performed deep sequencing of small RNAs (sRNAs) in *Brachypodium* as well as bread wheat and identified 70 known and 23 novel miRNAs. A diverse set of sRNAs was discovered in wheat plants subjected to either biotic (powdery mildew) or abiotic (high temperature) stress [16]. To ascertain whether miRNAs play any role in functioning of pollen mother cells during cold stress, sRNAs were sequenced from thermosensitive genic male sterile (TGMS) lines of wheat [17]. Out of 78 miRNA sequences identified in the study, 6 miRNAs were differentially expressed in cold stress in TGMS wheat lines [17]. In a recent study by Li et al. [18], sequencing of sRNAs along with degradome sequencing led to the identification of 32 miRNA families and confirmation of their targets.

Keeping in view the genome complexity as well as scarcity of information on hexaploid wheat miRNAs, the present study was performed to generate a comprehensive expression atlas of miRNAs in four tissues and three abiotic stress conditions by ultra-deep parallel sequencing approach combined with computational analyses. Using homology-based sequence analysis and publicly available miRNA prediction algorithms, we found that several of these miRNAs were conserved across many monocot species. A few miRNAs were detected and validated by quantitative PCR (qPCR) followed by their expression profiling in different tissues and abiotic stresses. Target genes of known as well as novel miRNAs were computationally predicted and gene ontology (GO) analysis was performed. Further, we validated the target genes of three known miRNAs using RLM-RACE and determined the expression levels of their predicted targets in various tissues/stress treatments. Mapping wheat sRNA sequences onto the genomes of closely related monocotyledonous plants highlighted extensive conservation of several miRNAs among these plants. To our understanding, this is the first comprehensive genome-wide study wherein miRNA profiling has been performed in four tissues and three abiotic stresses in wheat. We provide useful information on wheat miRNAs and their potential role in plant development and abiotic stress tolerance.

## Materials and Methods

### Plant growth and stress treatments

Wheat (*T. aestivum* cv. PBW343) seeds were surface-sterilized with 4% (v/v) sodium hypochlorite solution followed by 5–6 washes with sterile water and planted on muslin cloth for hydroponic growth under controlled conditions (temperature: 25±1°C; relative humidity: 70–75%; photoperiod: 16h light/8 h dark) in a growth room.

For the preparation of tissue-specific small RNA libraries, shoot and root tissues were collected separately from hydroponically grown seven-day-old seedlings. Mature leaf and spikelet were collected from field-grown plants 50 days after planting (DAP) and 90 DAP, respectively. Seven-day-old seedlings were employed for abiotic stress-related studies. For heat stress, seedlings were subjected to 35°C and 40°C for 30 min, 2 h, 4 h and 8 h each. Salinity stress was induced by treating the seedlings with 150 mM and 250 mM saline solution (sodium chloride) for 3 h, 6 h, 12 h and 24 h each. For water-deficit stress, seedlings were exposed to 20% PEG6000 (polyethylene glycol) and 400 mM mannitol for 1 h, 3 h, 6 h and 12 h each [19]–[22]. Tissues for all time-points of each stress were pooled to obtain four samples, which are referred to as: HS (high temperature stress), SS (salinity stress), WDS (water-deficit stress). A control sample (C) was kept as wheat seedlings grown under controlled conditions along with all the abiotic stress treatments.

For qRT-PCR experiments, tissues for all time-points were pooled for each agent/condition and eight samples thus obtained were designated as: HS_35, HS_40, SS_150, SS_250, WDS_PEG and WDS_MAN. For qPCR profiling of miRNAs in oxidative, hormone, cold stress and nutrient deprivation, seven-day-old hydroponically grown seedlings were subjected to the respective stress conditions. For oxidative stress, seedlings were treated with 10 µM methyl viologen (MV) and 10 mM hydrogen peroxide (H_2_O_2_) solution for 2 h and 4 h each. Response to several hormones was studied by treating seedlings with 1 µM brassinosteroid (BS), 50 µM gibberellic acid (GA), 100 µM methyl jasmonate (JA) and 10 µM abscisic acid (ABA) for 2 h and 4 h each. Seedlings were subjected to cold stress (CS) by placing at 4°C for 24 h and 72 h. Nutrient deficiency was mimicked by depriving nitrogen (N), phosphorus (P), potassium (K) and sulphur (S) for 72 h. All the harvested samples were immediately frozen in liquid nitrogen and stored at −80°C.

### RNA extraction, construction of small RNA libraries and deep sequencing

Total RNA was isolated from various tissues following a modified protocol by Chomczynski and Sacchi [23]. Lithium chloride method was employed for the enrichment of low molecular weight RNA (LMW) fraction [24]. Concentration of RNA was determined using spectrophotometer (Bio-Rad, USA) followed by quality analysis on MOPS-formaldehyde-agarose gel (total RNA) or TBE-urea-PAGE (LMW RNA).

For small RNA library construction, 50 µg of LMW RNA was resolved on 15% denaturing polyacrylamide gel and sRNAs in the size-range of 18–40 nucleotides (nt) were purified from the gel followed by sequential ligation with 5′ adapter and 3′ adapter. After each adapter ligation, size selection was performed using a polyacrylamide gel and ligated product was eluted from the gel. Subsequently, first strand cDNA was synthesized using Superscript II reverse transcriptase (Invitrogen, USA) and 3′ adapter-specific RT primer. The cDNA was amplified using adapter-specific sequencing primers and the amplified product was purified. Prior to sequencing, quality and quantity of the amplified small RNA-cDNA libraries was evaluated on Agilent 2100 Bioanalyzer system (Agilent Technologies, USA). Sequencing was performed using Illumina GAIIx sequencing platform at High-throughput Sequencing Facility, University of Delhi South Campus, New Delhi, India according to manufacturer's instructions (Illumina, USA). All the adapters, RT primer and sequencing primers were procured from Illumina, USA. The sequencing data was submitted to NCBI in gene expression omnibus (GEO accession no. GSE53487).

### Computational analysis of small RNA sequencing data

Purity filtered raw reads were analyzed using java-based UEA Small RNA Workbench version 2.4 [25]. Due to the unavailability of whole genome sequence of *Triticum aestivum*, sequence datasets from several resources (BACs, GSSs, ESTs available at NCBI and 5X coverage wheat genomic dataset available as EMBL/Genbank SRA accession number ERP000319) were compiled as ‘in-house wheat genome dataset’ to map putative sRNAs. UEA sRNA workbench v2.4 was employed for prediction of miRNAs and miRBase v20 database was used as a reference for identification of known and novel miRNAs. All putative miRNAs were further manually screened on the basis of criteria provided by Meyers et al. [26]. Briefly, following points were considered for miRNA prediction: (1) the mature miRNA and miRNA* (star strand of miRNA) sequence should be present in the opposite arms of the stem region of the hairpin structure with 2 nt overhangs at 3′ ends, (2) the predicted secondary structure should have lowest minimum free energy, (3) the secondary structure should not have more than 4 nt mismatches between miRNA and miRNA*.

To identify differentially expressed miRNAs in tissue-specific and abiotic stress-specific libraries, the tags or read counts were normalized. Normalization was carried out by dividing the number of reads with total number of putative small RNA population in each sample and multiplying by a million (10^6^). The obtained value was referred as TPM (tags per million). Fold change was calculated employing the formula: log_2_(treatment/control) [16].

### Validation and expression profiling of miRNAs and their target genes by quantitative PCR (qPCR)

PolyA tailing combined with qPCR method was employed for validating and evaluating the expression of predicted miRNAs [27]–[30]. For the extraction of total RNA, tissues of different time-points were pooled for each stress condition. Two µg of total RNA was polyadenylated using 1.5 U polyA polymerase enzyme (Ambion, USA) at 37°C for 1 h followed by purification using RNAeasy MinElute Cleanup Kit (Qiagen, Germany). Two µg of polyA-tailed RNA population was reverse transcribed using 1 µg RTQ primer and Superscript III reverse transcriptase (Invitrogen, USA) at 50°C for 1 h. Real-time PCR amplification was carried out using Mastercycler Realplex 2 (Eppendorf, Germany) with KAPA PROBE FAST Universal qPCR kit (KAPABiosystems, USA) according to manufacturer's instructions. Wheat 5S ribosomal RNA (GenBank accession #: FJ882478.1) was used as the endogenous control for quantification. Primer sequences employed for miRNA and miRNA* validation are presented in Table S8 in File S1.

Target prediction was performed employing psRNATarget (http://bioinfo3.noble.org/psRNATarget/) using default settings from wheat genome DFCI gene index (TAGI) release 12. To analyze the functional categories of targets, the blast2GO server was employed (http://www.blast2go.com). This server performs a homologous search against the GO database and resulting targets were further classified on the basis of their GO term enrichments. To validate the cleavage of the target by miRNA, a modified 5′ RLM-RACE was performed [31]. Initially polyA+ RNA was enriched from total RNA using PolyATtract mRNA Isolation System (Promega, USA). 25 ng of polyA+ RNA was ligated to 5′-RACE adapter and cDNA was prepared using random decamers. Two rounds of PCR were performed, first using the 5′-RACE outer primer and gene-specific outer primer followed by nested PCR employing 5′-RACE inner primer and gene-specific inner primer. Amplified products were gel purified, cloned in pGEM-T Easy vector (Promega, USA) and sequenced (Macrogen, Korea).

For the quantification of expression of putative target genes, 10 µg of total RNA was treated with DNase I (NEB, USA) and reverse transcribed using iScript cDNA synthesis kit as per manufacturer's instructions (Bio-Rad, USA). Target-specific primers were designed and amplification was performed using KAPA SYBR FAST Universal Master Mix (KAPABiosystems, USA) for real-time quantification. Wheat adenine phosphoribosyltransferase1 (APT: GenBank accession number: U22442.1) was employed as a reference gene for normalization. For all the qPCR experiments, three independent biological replicates were included. Fold-change was calculated using 2^−ΔΔCt^ method [32] and an average of three biological replicates was plotted along with calculated standard error (SE). Nucleotide sequences of primers employed for RACE and gene expression analysis are provided in Table S8 in File S1.

## Results and Discussion

The combinatorial approach of high-throughput sequencing and computational analysis of sequencing data has emerged as a gold standard for genome-wide identification of miRNAs in diverse organisms. Next generation sequencing based approaches have led to the discovery of small RNAs in plants on an unprecedented scale. Despite a few reports on miRNAs from hexaploid wheat, a comprehensive study on small RNAs from specific tissues as well as abiotic stresses is lacking. Herein, we present a detailed study on discovery of known as well as novel miRNAs in wheat in four tissues (including both vegetative: root, shoot, mature leaf and reproductive: spikelet) and in three abiotic stress conditions (high temperature, salinity and water-deficit stress).

### Sequence analysis of tissue-specific and abiotic stress-specific wheat small RNA libraries

**Figure 1:**
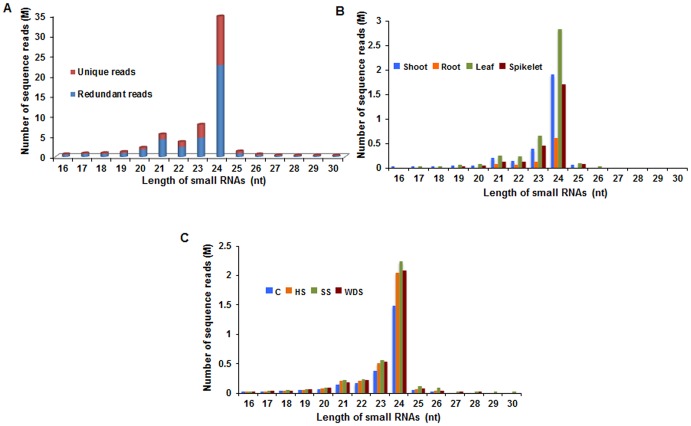
Length distribution of putative small RNA reads obtained by high-throughput small RNA sequencing of wheat (*Triticum aestivum*) libraries. (A) Reads were pooled from all the eight libraries and size distribution analysis was performed with redundant and unique reads. (B) Length distribution of unique reads obtained in tissue-specific libraries. (C) Length distribution of unique reads obtained in abiotic stress-specific libraries. C: control wheat seedlings; HS: high temperature stress; SS: salinity stress; WDS: water-deficit stress. Read counts are shown in millions (M).

To investigate the role of sRNAs in regulating abiotic stress response as well as development in wheat, eight small RNA libraries were generated using total RNA isolated from several wheat tissues and from seedlings that were exposed to different abiotic stresses. These libraries were subsequently sequenced using Illumina Genome Analyzer IIx platform. Sequencing reads of all the eight libraries were pooled resulting in a total of 59.5 million purity filtered reads which were analyzed using UEA sRNA workbench v2.4 [25]. Adapter trimming and removal of redundancy resulted in 32.5 million unique reads, which were then processed for removal of reads smaller than 16 nt and longer than 30 nt. The reads so obtained were cleaned resulting in elimination of sequences matching tRNAs, rRNAs, invalid sequences and low complexity sequences (simple sequence repeats or SSR, tandem repeats or TRs) (Table 1). A total of 20.3 million reads were retained as putative unique small RNA population (62.5% of the total unique population), which indicates good library quality and high depth of sequencing. Size distribution analysis of the redundant small RNA reads revealed that majority of reads (approximately 80%) were in the range of 20 to 24 nt, with 24 nt being the most abundant (55% of the total population) followed by 23 nt (11%) and 21 nt (10%) (Figure 1A). Unique reads showed somewhat similar abundance profile with 24 nt (59%) exhibiting the maximum representation followed by 23 nt (16%) and 21 nt (6%) (Figure 1A). We also compared the size distribution of putative sRNAs in tissue-specific and stress-specific libraries and found similar profile to that observed with the pooled dataset (Figure 1B, C). Overall, majority of sRNAs were in the size range of 21 to 24 nt, which is characteristic of Dicer-like (DCL)-processed sRNAs [33]. Previous studies on high-throughput sequencing of sRNAs in wheat and several other plants have reported similar observations with 21–24 nt class of sRNAs being the most prominent [16]–[18], [31], [34]–[38]. Amongst these, over-representation of the 24 nt sRNAs in all the libraries highlights the complexity of wheat genome as 24 nt heterochromatic small interfering RNAs (hc-siRNAs), generated from repeat regions and transposons, are known to maintain genome integrity by promoting heterochromatin formation [39]. The prominence of 23 nt population could be attributed to possible degradation of 24 nt sRNA species or small RNA length diversity [40]. Previous reports have shown that sRNAs of 23 nt were abundant in tomato fruit [41] and embryogenic calli of cotton [42]. Moreover two non-canonical sized sRNA species (22 to 26 nt and 23 to 27 nt) are generated from novel non-conserved MIR genes in *Arabidopsis*, rice and moss [6]. It would therefore be worthwhile to analyze these 23 nt and 22 nt long sRNA sequences found in wheat sRNA datasets in greater detail.

**Table 1:** Summary of sequencing reads and ouptput of each step of elimination pipeline employed for prediction of putative miRNAs in wheat.

Elimination category	Number of redundant reads	Number of unique reads
**Total sequence reads**	59541021	32574580
**Sequence length (<16 & >30 nt)**	15178457	11922873
**Low complexity sequences**	21131	6522
**Invalid sequences**	300657	229912
**rRNA/tRNA matches**	2455844	66367
**Putative small RNA population**	41584932	20348906
**Reads mapped onto wheat genome**	12062113	3673564

Pooled purity filtered reads obtained from small RNA sequencing of wheat libraries (control, high temperature stress, salinity stress, water-deficit stress, shoot, root, mature leaf and spikelets) and processed through an elimination pipeline (UEA sRNA workbench v2.4).

### Identification of known and novel miRNAs in wheat small RNA sequence dataset

In order to identify known and novel miRNAs in wheat, the putative sRNA population was processed with miRCAT tool of UEA sRNA workbench. Due to the unavailability of whole genomic sequence of *Triticum aestivum*, the unique population of putative small RNA sequences was mapped to a locally created ‘in-house wheat genome dataset’ containing sequences of BACs, GSS, ESTs and 5X coverage of NGS genomic dataset available at NCBI. More than 12 million putative small RNA sequences mapped perfectly onto reference dataset.

Mapped small RNAs were processed for retrieval of flanking precursor sequence (100 nt each from both ends) and RNA folding for secondary structure prediction (Figure S1). We were able to identify 47 known miRNAs (with sequence identical to miRBase v20 entries) and 1079 novel miRNAs (≥3 mismatches with miRBase entries). These 47 known miRNA sequences exhibited canonical hairpin loop structure and out of these only 8 miRNAs, namely miR156i-5p, miR164d, miR167, miR169, miR171, miR396a, miR396d and miR1432, were found to possess corresponding star sequences (also known as ‘3p sequence’) in the wheat putative sRNA population (Table S1 in File S1). The identified known miRNAs belonged to 20 families, out of which 9 (miR1117, miR1120, miR1135, miR1136, miR1318, miR1432, miR1436, miR5084 and miR6201) were monocot-specific [43]. To account for evolutionary divergence of known miRNAs in wheat, we predicted 46 sequences having homology to previously reported miRNAs in miRBase and these sequences were categorized as ‘variants of known plant miRNAs’ (Table S2 in File S1). While the length of predicted mature miRNAs was in the range of 19 to 24 nt, the length of predicted precursor sequences ranged from 60 to 217 nt, as reported in several previous studies [10] and GC content was between 19.76 and 87.5%, which correlated with those of previously reported miRNAs in plants [44]. High-throughput NGS technologies have enabled identification of many novel miRNAs even though they are present in low abundance. However when compared with earlier reports we found that the nucleotide sequences of 59 novel miRNAs were already reported by other groups [14], [16], [34], [45]. Since these 59 wheat miRNAs have not been deposited in miRBase v20, we included these miRNAs in the category of ‘novel miRNAs’ in this study.

Detection of star sequence of miRNAs in the sequencing dataset provides more authenticity to putative miRNAs. On the basis of precursor folding, star sequences of novel miRNAs were predicted and searched in the wheat small RNA dataset. Depending upon the detection of corresponding star sequence in our sequencing dataset, predicted novel miRNAs were further categorized as: (1) true novel wheat miRNAs, for which star sequence was present, were named as tae_x and (2) candidate novel wheat miRNAs, for which corresponding star sequence was not detected, were named as tae_Cx. Our analysis revealed 49 true novel (Table 2, Table S3 in File S1) and 1030 candidate novel miRNAs (Table S4 in File S1) in wheat. Presence of a relatively larger number of candidate novel miRNAs compared to true novel miRNAs is consistent with previous reports as miRNA-star molecules predominantly have low stability resulting in their reduced abundance [46], [47]. To our understanding, this is the first study wherein such an extensive sRNA profiling has been carried out for four different tissues and three variable abiotic stresses in a complex genome, and this probably explains identification of such a large number of miRNAs in wheat. Length distribution analysis of novel wheat miRNAs showed that most abundant sequences were 21 nt (777) in length followed by 22 nt (207) and 20 nt (95), which is in agreement with the observation that majority of plant miRNAs are 21 nt in length [31], [34], [35].

**Table 2:** List of identified true novel miRNAs, their star sequence and predicted targets in wheat.

miRNA	Sequence	Length	miRNA*	Target	Action
**tae_1**	AAACUAAUAUAUGAGCGUUUA	21	AAUGCUCUUAUAUUAGUUUAC	TC437556	C
**tae_2**	AAUUCGGGACGGAGGGAGUAA	21	ACUCCCUCCGUCCGGAAAUA	TC459057	C
**tae_3**	UUUCUGCGACGAGUAAUUCGG	21	AAUUACUCGUCGCAGAAAUGG	TC427972	C
**tae_4**	ACUUCCUCCGUUCGGAAUUAC	21	GUAAUUCCGAACGGAGGGAGUA	CV763629	C
**tae_5**	AGACAAGUAAUUUCGAACGGA	21	UCCGUUCGGAAUUACUUGUCG	TC390088	C
**tae_6**	AGCGAUCUGCCGAAGCUGUU	20	AUGCAUCGGUAGGGGAGCGU	FL645778	C
**tae_7**	AGGCCCACUGGGCAGCGCCCC	21	GCGCUGCCCUGUGAGCCUGC	CK160940	T
**tae_8**	AGUAAUUUUGGACGGAGGGAG	21	ACUCCCUCCGUCCGGAAUUA	CJ562562	C
**tae_9**	AUAAGCACCGGUGCUUAAGGA	21	CUGAAGCACCGGUGCCUAUUU	TC416715	C
**tae_10**	AUUAGUACUGGUUCGUGGCAC	21	GCCACGAACCGGUACUAAUGA	CA636114	C
**tae_11**	AUUUCUGGACGGAGGGAGUAA	21	ACUCCCUUCGUCCGGAAAUAC	BJ253379	C
**tae_12**	CAUCUAUUUUGGAACGGAGGG	21	UCCGUUCCAAAAUAGAUGACC	CK207929	C
**tae_13**	CCCUGGCAGAUAGCGCGAUCA	21	UGAACGGCACUUGCACAUGGG	TC402919	C
**tae_14**	CGCGCUGCCCUGUGAGCUUGC	21	AGGCCCACCGGGCAGCGCCC	TC400184	C
**tae_15**	CGGUAGGGCUGUAUGAUGGCGA	22	GCCAUCAUACGCCCAACCGUG	CJ881369	C
**tae_16**	UUUGACCAAGUUUGUAGAGAA	21	UUUUCUCUAUAAACUUGGUCA	CD452777	C
**tae_17**	CGGUUGGGCUGUAUGAUGGCGA	22	GCCAUCAUACGUCCAACCGUG	TC459829	C
**tae_18**	CUGACAUACGGGCGUGUGGGC	21	CGCCCACACGCACGCGUGUGAG	TC394522	C
**tae_19**	GGGCGUUCGCGCGGGCCGACC	21	UCGGCCUCGCGCUCGCCCUC	CA594286	C
**tae_20**	GGUGAACGCGCCGCCGUCAAAC	22	UUUGCCGGCGUGCGCGAGCACC	CA682386	C
**tae_21**	GGUGAGCGCGCCGCCGUCGAA	21	UUCGCCGGUCGCGCGUUCCCC	TC383147	T
**tae_22**	GUGCGCGGUCUGUUUUGGUCAG	22	GGCCGAGCGACGGACGGUGCCG	TC458773	C
**tae_23**	UAAUGUAAGACGCUUUUUGAC	21	UCAAAAAACGUCUUACAUUAU	CD881202	C
**tae_24**	UACCACGACUGUCAUUAAGCA	21	UGCUUAAUGACAGUCGUGGUG	AJ603583	C
**tae_25**	UAGCUCCACUAAAUUUGGAGCU	22	CUCCAAACUUAGUGAGCUAAG	CA698993	C
**tae_26**	UAUGGAUGAAGAUAUGCACUG	21	AGAAAGCAUUGUCUGACGUC	CJ882127	C
**tae_27**	UCCCGAAAGGCUUGAAGCAAAU	22	UAGCUUCAAGCCUUGAGGAAUA	TC435172	T
**tae_28**	UCUGAUUUACUCGUCGUGGUU	21	ACCACGACGAGUAAAUCGGAA	CD888727	C
**tae_29**	UCUGUAAACUAACAUAAGAGC	21	UCUUAUAUUAGUUUACGGAGG	No target	NA
**tae_30**	UCUGUAAACUAAUGUAAGAGC	21	UCUUAUAUUAGUUUACGGAGG	No target	NA
**tae_31**	UCUGUGACAAGUAAUUCCGAA	21	UCGGAAUUACUUGUCUCGGAU	CJ600810	C
**tae_32**	UCUUACAUUAUGGGACGGAGU	21	UCCGUCCCAUAAUGUAAGACG	TC433469	C
**tae_33**	UGAACGUGUGCUGAACGCGGA	21	UGCGUUCGGUACUUGAUCGG	CA683773	C
**tae_34**	UGACAAAUAUUUUCGGACGGA	21	CCUCCGUCCGGAAAUACUUG	CJ715057	C
**tae_35**	UGACAACUAUUUUCGGACGGA	21	UCCGUCCGGAAAUACUUGUCA	BJ276292	C
**tae_36**	UGACAAGUACUUUCGGACGGA	21	UCCGUCCGGAAAUACUUGUCA	BJ276292	T
**tae_37**	UGACAAGUAUUCUCGGACGGA	21	UCCGUCCGGAAAUACUUGUCA	TC433218	C
**tae_38**	UGACAAGUAUUUCGGACGGA	20	UCUGUCCGGAAAUACUUGUCA	BJ276292	T
**tae_39**	UGACAAGUAUUUUCGAACGGA	21	UCCGUCCGGAAAUACUUGUCA	BE423820	C
**tae_40**	UGAUAAGUAUUUUCGGACGGA	21	UCCGUCCGGAAAUACUUGUCA	BJ276292	C
**tae_41**	UGCAGUGGCAUAUGCAACUCU	21	AGAGCUGCAUUUGCACCUGCA	GH730467	C
**tae_42**	UGGCGAGGGACAUACACUGU	20	UACAGUUUAUGUCCCCGGCAG	GH730467	C
**tae_43**	UUCCGAAAAGCUUGAAGCAAAU	22	UAGCUUCAAGCCUUGAGGAAUA	TC435172	T
**tae_44**	UUCCGAAAGGCUUGAAGCAAAU	22	UAGCUUCAAGCCUUGAGGAAUA	CA647790	C
**tae_45**	UUCCGAAAGGCUUGAAGCGAAU	22	UAGCUUCAAGCCUUGAGGAAUA	DR735126	C
**tae_46**	UUCGAUCGUAAUCGGAUGGUC	21	GGAUGAUCCGGACACGACGGU	CA678208	C
**tae_47**	UUCUGAAAGGCUUGAAGCAAAU	22	UAGCUUCAAGCCUUGAGGAAUA	CD921947	C
**tae_48**	UUGUCUUAGAUUCGUCUAGAUA	22	UAUUUAGACAAAUCUAAGACA	TC439689	C
**tae_49**	UUUCGAAAGGCUUGAAGCAAAU	22	UAGCUUCAAGCCUUGAGGAAUA	TC427740	C

Accession numbers of predicted target are presented along with the possible mode of action of miRNA on the target. miRNA*: star strand of miRNA; C: mRNA cleavage; T: translational repression; NA: not applicable as no target could be predicted.

**Figure 2:**
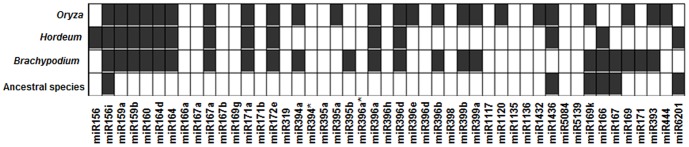
Conservation of identified known miRNAs in wheat among other related monocot species. Square boxes with highlighted grey color indicate the presence of miRNA in the related monocot species such as ancestral species (*Triticum urartu*, *Aegilops speltoides* and *Aegilops tauschii*), *Brachypodium distachyon, Hordeum vulgare* and *Oryza sativa.*

We also checked for the level of conservation of known wheat miRNAs in other monocots such as *Brachypodium distachyon*, *Oryza sativa*, *Hordeum vulgare* and few progenitors of wheat (*Aegilops tauschii*, *Aegilops speltoides* and *Triticum urartu*), the results of which are presented in Figure 2. miR156i is evidently present in all the species included in this study indicating a high degree of conservation among several monocotyledonous plants. While maximum number (24) of miRNAs matched with rice miRNAs, only six known miRNAs were found to match with the more closely related wheat ancestral species. This difference could be attributed to the vast amount of resources available in rice such as its complete genome sequence in addition to huge number of research reports on identification of miRNAs in rice as compared to limited information on the ancestral species.

### Expression profiling of known and true novel miRNAs in different tissues of wheat

Differential expression of miRNAs, spatially, temporally or conditionally, is a reliable parameter for predicting their physiological functions and can be analyzed using ‘read number’ in the high throughput sequencing data. We compared the expression levels of known as well as novel miRNAs identified in this study in different tissues and during abiotic stresses. While the absolute abundance of known miRNAs reached upto 531648 reads (zma-miR156i-5p), novel miRNAs exhibited a maximum of 31947 reads for tae_C898. Lower abundance of novel miRNAs when compared with known miRNAs has previously been reported and is attributed to their species-specificity and recent ‘birth and death of MIRNA genes’ [16], [48], [49]. Amongst known miRNAs, MIR156 was the most abundant family consisting of 534830 tags followed by MIR166 with 14643 counts, whereas MIR1120 was the least abundant with only 1 read count as shown in Table S1 in File S1. Meanwhile tae_C898 with 31947 read counts followed by tae_24 with 22653 read counts were the two most abundant novel miRNAs (Table S3, S4 in File S1).

**Figure 3:**
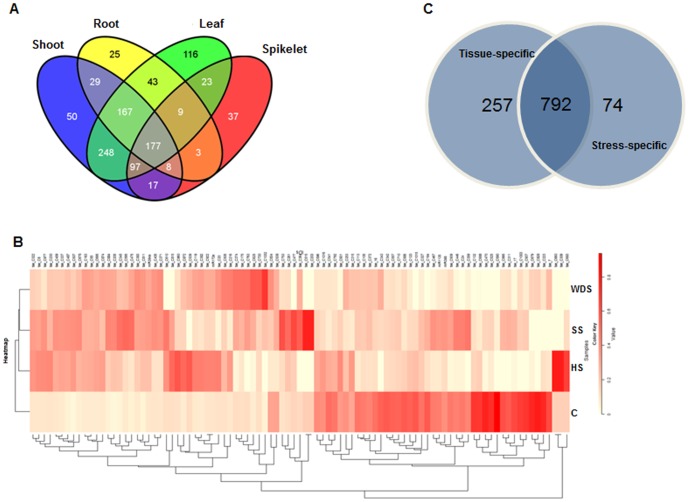
Analysis of digital expression of miRNAs in wheat tissue- and abiotic stress-specific libraries. (**A**) Venn diagram representing overlap of miRNA population in tissue-specific libraries viz. shoot, root, mature leaf and spikelet. (**B**) Unsupervised hierarchical cluster analysis of the normalized expression levels (TPM) of miRNAs during three abiotic stresses (high temperature, salinity and water-deficit stress). The clustering of three stress-treated samples along with control sample was performed using Pearson uncentered algorithm with an average linkage rule, to identify clusters of miRNA based on their expression levels across samples. miRNAs exhibiting ≥2 fold change in expression levels were included in this analysis. (**C**) Venn diagram representing small RNAs overlapping among tissue- and stress- specific libraries. C: control wheat seedlings; HS: high temperature stress; SS: salinity stress; WDS: water-deficit stress.

Spatio-temporal regulation of miRNAs plays a crucial role in plant growth and development. To identify miRNAs that are exclusively expressed as well as those displaying overlapping expression in different tissues, digital gene expression of known as well as novel miRNAs was studied among these tissues. While 177 miRNAs were expressed in all the four tissues, 50, 25, 116 and 37 miRNAs were specific to shoot, root, mature leaf and spikelet, respectively (Figure 3A). Significantly, a higher number of miRNAs (248) was expressed both in shoot and mature leaf. Only three miRNAs could be detected in both root and spikelet, which is expected on the basis of their weak spatio-temporal and morpho-physiological relationships (Figure 3A and Table S1, S3, S4 in File S1). Unsupervised hierarchical clustering of 95 miRNAs exhibiting ≥ 2-fold change in seedlings exposed to three abiotic stresses with respect to control seedlings in normalized digital gene expression analysis revealed that several known as well as novel miRNAs were regulated by abiotic stresses. The resulting dendrogram shows miRNAs clustering across different stress-specific wheat sRNA libraries (Figure 3B and Table S1, S3, S4 in File S1). Hierarchical clustering on the basis of expression of miRNAs confirms that molecular changes induced by salinity stress and water-deficit conditions are largely similar as they are clustered together. This observation is in accordance with previous studies indicating that similar genes are involved in both pathways [50]–[52]. Additionally, molecular components of some of the stress-regulated and developmental pathways are known to interact [53], [54] and our data supports this observation as 792 miRNAs were expressed in both stress-specific and tissue-specific libraries (Figure 3C). Many previous reports have indicated that miRNAs play a major role in controlling developmental aspects in plants [55]. Our analysis is in agreement as a larger number of miRNAs (257) expressed in a tissue-specific manner as compared to those expressed under abiotic stress (74).

**Figure 4:**
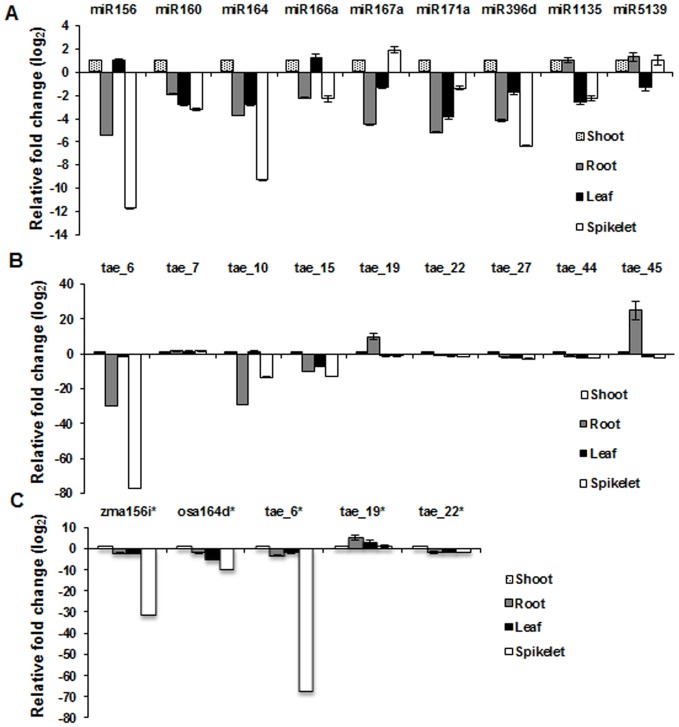
Tissue-specific expression profiling of miRNAs identified in wheat small RNA libraries by qPCR method. (**A**) known miRNAs, (**B**) true novel miRNAs, (**C**) miRNA* sequences. PolyA tailing of total RNAs followed by cDNA synthesis and Taqman-based qPCR was employed for validation of miRNAs and normalization was carried out with wheat 5 S rRNA. Error bars represent standard error of three independent biological replicates.

To further validate some of the predicted miRNAs in wheat, qRT-PCR was employed to measure their expression levels in different tissues. We analyzed the expression pattern of 9 known miRNAs (miR156, miR160, miR164, miR166a, miR167a, miR171a, miR396d, miR1135 and miR5139) and 9 true novel miRNAs (tae_6, tae_7, tae_10, tae_15, tae_19, tae_22, tae_27, tae_44 and tae_45) (Figure 4 and Figure S2). For tissue-specific expression profiling shoot tissue of seven-d-old seedlings was taken as control against which miRNA expression changes were compared in various tissues (root, mature leaves and spikelet). While 7 of the 9 known miRNAs tested (miR156, miR160, miR164, miR166a, miR167a, miR171a, miR396d) were down-regulated in root tissues, expression of miR1135 and miR5139 was comparable in root and shoot tissues. In *Arabidopsis*, MIR164 family members act redundantly during shoot development [56] and expression of miR164 in shoot tissue is indicative of its physiological role in wheat shoot development. Levels of 7 known miRNAs, except miR167a (which showed ∼2 fold accumulation) and miR5139 (which remained unaltered), were significantly lower in spikelet as compared to their expression in shoot tissue (Figure 4A). Previous studies have advocated the role of miR167 in flowering in *Arabidopsis* [57] and grain development in rice [58] and its enrichment in wheat spikelet indicates towards its importance in reproductive development in wheat. Two of the miRNAs, miR156 and miR166a, expressed at significantly high levels in mature leaf (Figure 4A) which is in agreement with earlier reports implicating the role of miR156 in phase change and leaf development [59], [60] and miR166 in establishing leaf polarity [61]. To our understanding this is the first report displaying tissue-specific expression of two miRNAs, miR1135 and miR5139, in plants. miR5139 was first detected in a perennial herb, *Rehmannia glutinosa*, but no function was assigned to this miRNA [62] and till date no other plant species has been reported to contain a homolog of miR5139. Recently, miR1135 was located on the short arm of chromosome 5D of wheat and a similar sequence was found in *B. distachyon* also [63].

Among the nine true novel miRNAs examined, two miRNAs (tae_19 and tae_45) were highly abundant in root tissue, which indicates that these miRNAs might be involved in root growth and development (Figure 4B). The remaining six novel miRNAs showed down-regulation in root tissue, of which tae_6, tae_10 and tae_15 exhibited approximately 10-fold decrease in the expression levels. tae_7 did not show significant change in expression levels between root and shoot tissue. Seven novel miRNAs, except tae_7 and tae_10, showed reduced expression levels in mature leaf as compared to shoot tissue. All miRNAs tested in this study, except tae_7, exhibited decline in their expression levels in spikelet with respect to shoot. A few reports have indicated that star strand of miRNAs participate in regulating biological processes [64], [65]. Moreover the detection of star strand of miRNA provides additional authenticity to their corresponding main strand of miRNA. We therefore performed profiling of star sequences of 5 predicted miRNAs and found similar pattern of expression when compared with that of their corresponding main strand (Figure 4C, Figure S2). To further elucidate the specific role of these miRNAs in plant development, it is pertinent to perform an expression kinetic study with different stages of development for a particular tissue.

### Expression profiling of known and true novel miRNAs in wheat seedlings exposed to different abiotic stresses

**Figure 5:**
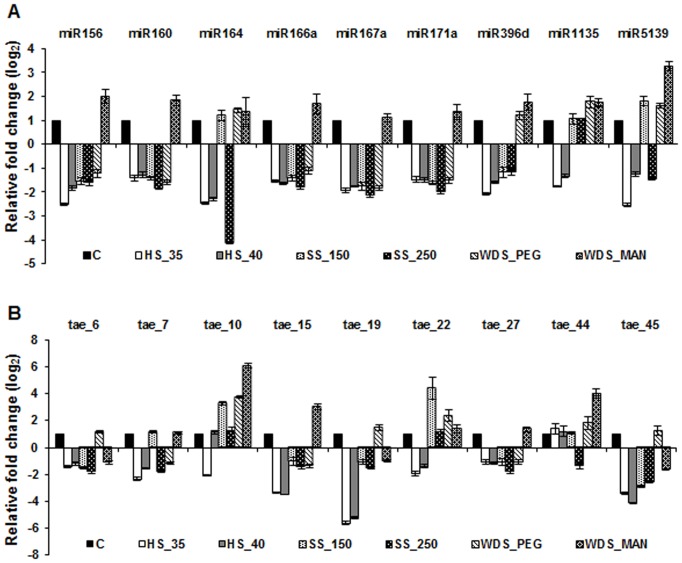
Abiotic stress-specific expression profiling of miRNAs identified in wheat small RNA libraries by qPCR method. (**A**) known miRNAs, (**B**) true novel miRNAs. PolyA tailing of total RNA followed by cDNA synthesis and Taqman-based qPCR was employed for validation of miRNAs and normalization was carried out with wheat 5S rRNA. Error bars represent standard error of three independent biological replicates. C: control wheat seedlings; HS_35: high temperature stress at 35°C; HS_40: high temperature stress at 40°C; SS_150: salinity stress with 150 mM NaCl; SS_250: salinity stress with 250 mM NaCl; WDS_PEG: water-deficit stress imposed by 20% PEG; WDS_MAN: water-deficit stress imposed by 400 mM mannitol.

Several miRNAs have been reported to be involved in defining plant response against abiotic stress [8], [66], [67]. To investigate whether some of the identified miRNAs are differentially regulated by abiotic stresses, expression profiling was performed in seedlings exposed to high temperature, salinity and water-deficit stress. Seedlings grown in controlled conditions were included as control and for calculating relative expression levels. All the known miRNAs that were studied showed down-regulation, with miR156, miR164 and miR5139 exhibiting more than 2-fold change, in response to high temperature stress (Figure 5A). In case of all novel miRNAs, except tae_6, tae_27, tae_44 (which were largely unaltered), all the other (tae_7, tae_10, tae_15, tae_19, tae_22, tae_45) miRNAs were down-regulated under high temperature conditions (Figure 5B). The most significant decline (>3-fold change) in expression was observed for tae_15, tae_19 and tae_45. Similarly all the known miRNAs (except miR1135 which showed no change) were slightly down-regulated when seedlings were exposed to salinity stress. Interestingly, levels of miR164 that remained unchanged in response to 150 mM NaCl solution displayed more than four-fold decline when 250 mM NaCl of salt solution was applied hydroponically. Under similar conditions, a contrasting response was observed for miR5139 whose expression level increased 1.8-fold at 150 mM NaCl and decreased 1.45-fold when higher concentration (250 mM) of NaCl was applied. The majority of novel miRNAs (tae_6, tae_15, tae_19, tae_27 and tae_45) displayed down-regulation in response to salinity stress (Figure 5B) with tae_45 showing maximum (more than two-fold) decline. The expression levels of two novel miRNAs i.e., tae_10 and tae_22 increased substantially in response to 150 mM salt stress. Noticeably, expression of tae_7 and tae_44 (that remained unaltered with the application of 150 mM NaCl) decreased on exposure of seedlings to 250 mM NaCl. Water deficiency stress was imposed by exposing seedlings to either PEG or mannitol. We found that miR156, miR160, miR166a, miR396d, miR1135, miR5139, tae_10, tae_15 and tae_44 exhibited approximately two-fold induction in expression levels when mannitol-induced water-deficiency stress was imposed (Figure 5A, B). This finding corroborates other reports wherein several of these miRNAs have been shown to be drought stress-responsive in *Arabidopsis*, rice and *T. dicoccoides* [13], [66], [68]. Surprisingly, out of 18 miRNAs tested in this study, 11 miRNAs (miR156, miR160, miR166a, miR167a, miR171a, tae_6, tae_7, tae_15, tae_19, tae_27 and tae_45) exhibited contrasting expression profile in seedlings exposed to mannitol as compared with the seedlings stressed with PEG (Figure 5A, B). This response could possibly be attributed to different modes of action and/or the associated toxic effects of these agents [69], [70]. It is noteworthy that miR5139, tae_10, tae_22 and tae_44 were significantly up-regulated in response to both water-deficit agents. Target validation and their expression profiling under different abiotic stress conditions would help in the identification of novel components in stress-responsive pathways in wheat.

**Figure 6:**
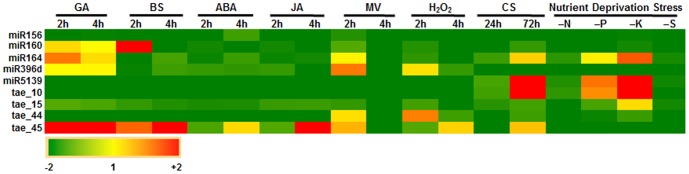
Heat map representing q-PCR based expression profiling of wheat miRNAs during cold stress, nutrient stress, oxidative stress and exogenous application of various hormones. PolyA tailing of total RNA followed by cDNA synthesis and Taqman-based qPCR was employed for validation of miRNAs and normalization was carried out with wheat 5 S rRNA. GA: gibberellic acid; BS: brassinosteroids; ABA: abscisic acid; JA: methyl jasmonate; oxidative stress imposed by hydrogen peroxide (H_2_O_2_) and methyl viologen (MV); CS: cold stress; N: nitrogen deprivation; P: phosphorus deprivation; K: potassium deprivation; S: sulphur deprivation.

Significant changes in the expression pattern of both known and novel miRNAs under three abiotic stresses prompted us to determine the levels of these miRNAs in response to other abiotic stresses such as cold stress (4°C), oxidative stress (imposed by either methyl viologen or hydrogen peroxide) and nutrient deprivation. Additionally, expression changes for these miRNAs were also recorded after exogenous application of plant hormones such as gibberellic acid (GA), abscisic acid (ABA), brassinosteroids (BS) and jasmonic acid (JA). Five known and four novel miRNAs were included in this study and we found that majority of these miRNAs were down-regulated under the imposed conditions (Figure 6). Exceptionally however, levels of miR160 increased significantly in response to application of BS. Two of the miRNAs (miR5139 and tae_10), were also inducible by prolonged (72 h) low temperature stress. A minor increase in the level of tae_44 was observed when hydrogen peroxide was applied to seedlings. tae_45 displayed up-regulation in response to the application of GA, BS and JA. Two of the miRNAs- miR5139 and tae_10 were significantly up-regulated in response to both phosphorus and potassium deprivation. However, miR164 showed enhanced expression levels only when seedlings were exposed to potassium-deficient condition. Based on all the expression profile studies, it is undoubtedly clear that these miRNAs are modulated both by developmental and environmental cues and there is an intricate association between phytohormone signaling, plant development and abiotic stress responses, the key components of which are potentially regulated by miRNAs [71]. Overall, we highlight potential role of several known and novel miRNAs in plant development and/or regulation of abiotic stress in wheat.

### Mapping of putative wheat sRNA sequences onto other monocot genomes and their expression analysis

The unavailability of complete genome sequence in wheat is largely responsible for limited discovery of novel miRNAs in this crop plant. In order to harness additional information from wheat sRNA sequencing dataset generated in this study, the unique putative sRNA population was also mapped onto nucleotide database of other monocotyledonous plant species that included: 1) monocots related to wheat such as *Brachypodium distachyon*, *Hordeum vulgare* and *Oryza sativa*; 2) wheat ancestral counterparts (also mentioned collectively as “ancestors” hereafter) such as *Triticum urartu* (A genome donor of allohexaploid wheat), *Aegilops speltoides* (similar to B genome donor) and *Aegilops tauschii* (D genome donor). The miRNAs mapping onto ancestral genomes identified using this approach have been referred to as ‘anc-miRNAs’ throughout this paper. We were able to identify 449, 1002, 343 and 123 miRNAs in *Brachypodium*, *Hordeum*, *Oryza* and ancestors, respectively. Further analysis was carried out for only a subset of novel miRNA entries mapping onto different monocot species, which were categorized either as ‘true novel’ or ‘candidate novel’ as previously described. The number of novel miRNAs mapping onto genomic sequence of *Brachypodium, Hordeum, Oryza* and ancestors were 391, 912, 267, and 117 respectively (Table 3). Though none of the above discovered miRNAs was common to all the closely related species, 62 were similar between wheat and *Hordeum*. Significantly, 31, 18 and 16 wheat miRNAs were also found to be present in ancestors, rice and *Brachypodium* respectively (Figure S3). Details of miRNAs found in related monocot species are presented in Table S5.1-S5.4 in File S1. The above survey has uncovered the extent of conservation and diversity of novel miRNAs in monocot plant species. In the future, meaningful insights on the evolution of miRNAs and their precursors can be derived from the data generated in this study.

**Table 3:** Summary of mapping of wheat sRNA reads in related monocot species.

miRNAs	Ancestral Species	*Brachypodium*	*Hordeum*	*Oryza*
Known miRNAs	6	58	90	76
True Novel miRNAs	4	28	95	17
Candidate Novel miRNAs	113	363	817	250

Putative wheat miRNAs were mapped onto available genome sequence of other monocot plants (*Brachypodium distachyon*, *Hordeum vulgare* and *Oryza sativa*) and progenitors of wheat (*Triticum urartu*, *Aegilops speltoides* and *Aegilops tauschii*). Novel miRNAs for which corresponding star sequences were found (true novel); not found (candidate novel).

**Figure 7:**
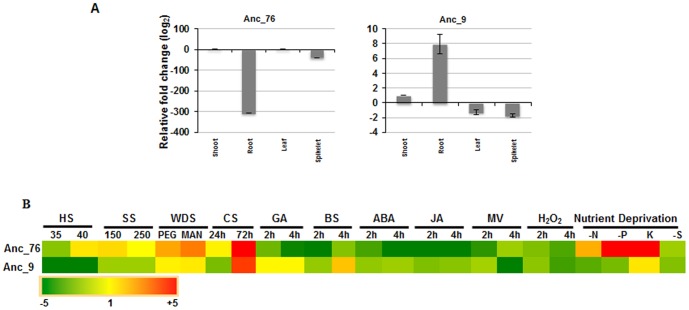
qPCR validation and expression analysis of wheat miRNAs mapping onto genomic sequence of ancestral species in tissues and during various stresses in wheat. PolyA tailing followed by cDNA synthesis and qPCR was employed for validation of miRNAs and the expression level of miRNAs were normalized with respect to wheat 5(A) qPCR of miRNAs in tissues; (B) Heat map representation of qPCR of miRNAs during abiotic stress and application of hormones. (HS_35: heat stress at 35°C; HS_40: heat stress at 40°C; SS_150: salinity stress with 150 mM NaCl solution; SS_250: salinity stress with 250 mM NaCl solution; WDS_PEG: water-deficit stress by 20% PEG solution; WDS_MAN: water-deficit stress by 400 mM mannitol; GA: gibberellic acid; BS: brassinosteroids; ABA: abscisic acid; JA: methyl jasmonate; oxidative stress imposed by hydrogen peroxide (H_2_O_2_) and methyl viologen (MV); CS: cold stress; N: nitrogen deprivation; P: phosphorus deprivation; K: potassium deprivation; S: sulphur deprivation)

Two of the miRNAs mapping onto ancestral genomes, Anc_9 and Anc_76, were experimentally validated by determining their expression profile in different tissues, under various abiotic stress conditions and in response to exogenous treatment of hormones. While Anc_9 mapped onto both wheat (named as wheat candidate novel miRNA, tae_C713) and *A. speltoides* genomic sequences, Anc_76 matched only with genomic sequence of *A. speltoides*. When compared with shoot tissue, Anc_9 was highly up-regulated (8-fold) while Anc_76 was drastically down regulated (306-fold) in root tissue (Figure 7A). Interestingly, Anc_76 was found to be up-regulated in response to water deficit stress and conditions of nutrient deprivation (potassium and phosphorous-deficiency). Both miRNAs exhibited significant accumulation when seedlings were treated with 72 h of cold stress (Figure 7B). It would be worthwhile to perform comparative genomic studies on these candidate miRNAs and their corresponding targets among various progenitors of wheat.

### Identification, validation and expression profiling of target genes of known and novel miRNAs

**Figure 8:**
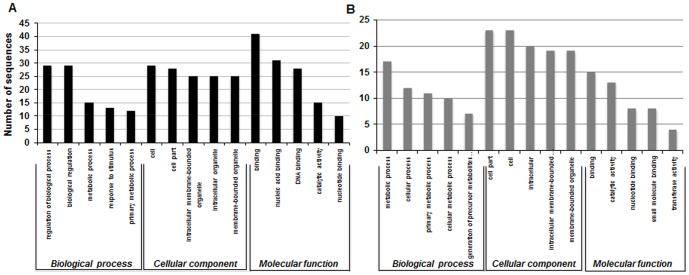
GO analysis of predicted targets of known and novel miRNAs. Top 5 sequences were selected for each category. Gene ontology of putative targets of (**A**) known miRNAs and (**B**) novel miRNAs.

Identification of gene(s) that are targeted by miRNAs is crucial for elucidating their biological function in plants and unraveling the complex regulatory network of miRNA-target interaction. Since plant miRNAs and their corresponding targets exhibit high sequence complementarity, target genes can be computationally predicted. Plant small RNA target finder (psRNA Target finder; www.palntgrn.org/psRNATarget/) software [72] was employed with default settings and wheat DFCI gene index (TAGI) version 12 was used as reference genome dataset. We predicted 402 and 8344 targets for 47 known and 1079 novel miRNAs in wheat, respectively (Table 2, Table S6.1–S6.3 in File S1). Except bdi-miR171c, tae-miR1136 and hvu-miR6201, which had only a single target all the other known miRNAs had multiple targets. Similarly, one target each was predicted for 3 novel miRNAs and 59 candidate novel miRNAs (Table S6.1–S6.3 in File S1). The maximum numbers of targets were 46 in case of MIR164 family and 24 for tae_16. No target genes could be predicted for miR166, miR1117, miR5139, tae_29 and tae_30, which could be due to unavailability of complete wheat genome sequence and annotation. One of the interesting targets for heat stress down-regulated tae_19 is the A6N0C7 Cluster: Polyubiquitin containing 7 ubiquitin monomers (TC392334). Polyubiquitin genes are induced under conditions of heat shock [73] and hence tae_19 could be a regulatory component of heat-induced protein surveillance machinery. Similarly, expression level of tae_22 also declined during heat stress for which one of the predicted targets is Q8H4Q9 Cluster: GTP-binding protein Rab6. The predicted target belongs to superfamily of GTP-binding proteins that are known to be involved in regulating diverse cellular processes, including stress tolerance in plants [74]. The predicted targets of both the known and novel miRNAs were subjected to gene ontology (GO) clustering analysis and it was found that maximum number of targets were associated with cellular component followed by molecular function (Figure 8, Table S7.1–S7.2 in File S1). Targets of several miRNAs are predicted to possess nucleotide-binding activity, are therefore, in all likelihood, represent transcription factors. Based on previous studies it is believed that plant miRNAs have strong propensity of targeting genes that encode for transcription factors [75]. In conclusion, the categorization of putative targets by GO analysis revealed that these miRNAs could be involved in diverse biological processes and varied physiological traits in wheat.

**Figure 9:**
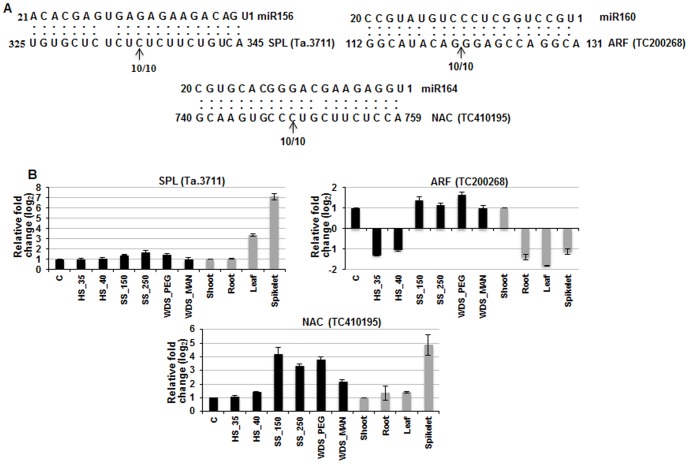
Validation of miRNA-directed cleavage of predicted target mRNA using 5′ RLM- RACE. (**A**) The cleavage sites of three target genes were identified using 5′ RLM-RACE analysis. The arrows indicate 5′ termini of miRNA-guided cleavage products and the number indicates frequency of the cloned PCR products sequenced. (**B**) Tissue- and abiotic stress-specific expression profiling of validated targets of wheat miRNAs. qPCR was performed and normalization was carried out with wheat APT (Adenine Phosphoribosyl Transferase) gene. Error bars represent standard error of three biological replicates. C: control wheat seedlings; HS_35: high temperature stress at 35°C; HS_40: high temperature stress at 40°C; SS_150: salinity stress with 150 mM NaCl; SS_250: salinity stress with 250 mM NaCl; WDS_PEG: water-deficit stress imposed by 20% PEG; WDS_MAN: water-deficit stress imposed by 400 mM mannitol.

On the basis of extent of sequence complementarity between miRNAs and putative target genes, target prediction software indicates the possible mechanism of action of miRNA as mRNA degradation or translational repression (Table 2, Table S5.1–S5.3 in File S1) [76]. A majority of plant miRNAs regulate expression of their target genes by cleaving the mRNA between 10 and 11 position with respect to the miRNA sequence [77]. To authenticate the cleavage of the target genes by the action of miRNAs, we performed a modified 5′ RLM-RACE experiment. 5′ RACE was performed, target cleavage products were cloned and sequenced to determine the precise mRNA cleavage sites. miR156, miR160 and miR164 were found to target wheat homologs of *A. thaliana* SPL gene, ARF10 and NAC1 [16], [58], [78]–[80]. It was found that all three known miRNAs tested in this study predominantly cleaved at the expected 10/11 position (Figure 9A). Additionally, we performed expression profiling of these target genes in various tissues and during stress conditions (Figure 9B). The targets of miR156 and miR164 were significantly up-regulated in spikelet tissue, which correlates inversely with the expression levels of corresponding miRNAs in the same tissue. Auxin response factor (ARF), target of miR160, did not exhibit any significant change in expression levels in response to the abiotic stresses as well as four tissues, which is in accordance with the minor changes in the levels of miR160 (Figure 4A, 9B). It is noteworthy that these targets belong to a large gene family and therefore it is important to determine how many members of each gene family are targeted by one miRNA. Previous studies have indicated that the same miRNA can target two different members of a family in a spatio-temporal manner [81]. Therefore it is equally important to determine the right pair (of miRNA and target) as well as condition (in which the target is cleaved by miRNA) to associate miRNA with a trait. In *Arabidopsis*, 16 members of plant specific SPL TF family involved in diverse developmental processes have been identified and of these 10 are targeted by MIR156/157 family [82]. Our analysis revealed that at least three wheat SPL genes exhibiting homology with rice SPL2, 11 and 16 are potentially targeted by miR156 which is in agreement with studies wherein 11 out of 15 SPL members were found to contain sequence complementary to MIR156 [83]. It is worthwhile to experimentally determine the target specificity of miR156 in wheat. Similarly, rice is known to encode 25 and 151 members of ARF and NAC family, respectively [84], [85]. More than one member of wheat ARF and NAC family was targeted by miR160 and miR164, respectively [86]. Identification of more members of SPL, ARF and NAC family of transcription factors in wheat would help in delineating more targets of miR156, miR160 and miR164, respectively and further provide insights on their role in plant development. Moreover, it is possible that different members are involved in different physiological processes and this could account for few tissues/stress conditions exhibiting inverse correlation in expression of miRNA and corresponding target gene. Manipulating the expression level of particular miRNA followed by studies on genome-wide changes would possibly provide clues on the potential targets. It would be interesting to experimentally validate targets for known as well as novel miRNAs identified in this study. To gain insight into miRNA-target interaction, high-throughput degradome sequencing of wheat tissue as well as stress-specific libraries when correlated with small RNA expression data will contribute to our understanding on the role of miRNAs and their targets in development and stress tolerance in wheat.

## Conclusions

We employed a combinatorial approach of high-throughput sequencing followed by computational prediction of miRNAs to identify 47 known, 49 true novel and 1030 candidate novel miRNAs in wheat. Tissue-specificity and stress-responsiveness of a few of these miRNAs was determined using qPCR method. Target genes of wheat miRNAs were predicted and GO analysis was performed to functionally group these genes. We further validated the target genes: SPL-like, ARF10 and NAC1 of wheat miR156, miR160 and miR164, respectively by RLM-RACE method. Expression profiling of the target genes revealed an inverse correlation with miRNA expression profile in some tissues and/or stress. Mapping of wheat small RNA reads onto genomic sequences of related monocot species resulted in identification of several known miRNAs. It would be worthwhile to prepare a global expression atlas of all the identified wheat miRNAs by customized microarray methodology and perform degradome sequencing of wheat libraries to validate target regulation by specific miRNAs. With the completion of wheat genome sequencing project followed by annotation, a huge increase in identification of novel miRNAs is expected. Our present study has generated a valuable resource on wheat miRNAs, which could be utilized not only for understanding stress responses but also for engineering effective stress tolerance in wheat.

## Supporting Information

Figure S1 **Schematic workflow for the identification of wheat miRNAs in high throughput sequence reads obtained with eight pooled wheat small RNA libraries.**
(TIF)

Figure S2 **Tissue-specific expression profiling of miRNAs identified in wheat by qPCR method.** PolyA tailing of total RNAs followed by cDNA synthesis and Taqman-based qPCR was employed for validation of miRNAs and normalization was carried out with wheat 5 S rRNA. Error bars represent standard error of three independent biological replicates.
(TIF)

Figure S3 **Overlap of miRNAs among wheat and related monocot species.** Venn diagram representing overlap of novel miRNA population mapping onto genomic sequences of wheat, ancestral species (*Triticum urartu*, *Aegilops speltoides* and *Aegilops tauschii*), *Brachypodium*, *Hordeum* and *Oryza*.
(TIF)

File S1 **File S1 includes the following: Table S1.** List of identified known microRNAs in wheat, their precursor information and read counts in each library. **Table S2.** List of identified variants of known microRNAs in wheat, their precursor information and abundance. **Table S3.** List of identified true novel microRNAs in wheat, their precursor information and normalized read counts in each library. **Table S4.** List of identified candidate novel microRNAs in wheat, their precursor information and normalized read counts in each library. **Table S5.1.** List of wheat miRNA sequences mapping onto genome sequence of ancestral species (*Aegilops speltoides, Triticum urartu* and *Aegilops tauschii*). The table contains precursor information along with the total abundance of miRNA sequence. **Table S5.2.** List of wheat miRNA sequences mapping onto genome sequence of *Brachypodium distachyon*. The table contains mapping summary, precursor information along with the total abundance of miRNA sequence. **Table S5.3.** List of wheat miRNA sequences mapping onto genome sequence of *Hordeum vulgare*. The table contains mapping summary, precursor information along with the total abundance of miRNA sequence. **Table S5.4.** List of wheat miRNA sequences mapped onto genome sequence of *Oryza sativa*. The table contains mapping summary, precursor information along with the total abundance of miRNA sequence. **Table S6.1.** Comprehensive list of predicted targets of identified known miRNAs in wheat. **Table S6.2.** List of predicted targets of true novel miRNAs in wheat. **Table S6.3.** Predicted targets of candidate novel miRNAs in wheat. **Table S7.1.** Gene ontology analysis of predicted targets of known miRNAs in wheat. **Table S7.2.** Gene ontology analysis of predicted targets of novel miRNAs in wheat. **Table S8.** Details of the primers employed in this study.
(XLS)
