# Supplementary figures and images for: A Comprehensive Genome-Wide Study on Tissue-Specific and Abiotic Stress-Specific miRNAs in Triticum aestivum
Source: PLoS One. 2014 Apr 23;9(4):e95800. doi: 10.1371/journal.pone.0095800 (PMC3997425; doi:10.1371/journal.pone.0095800)

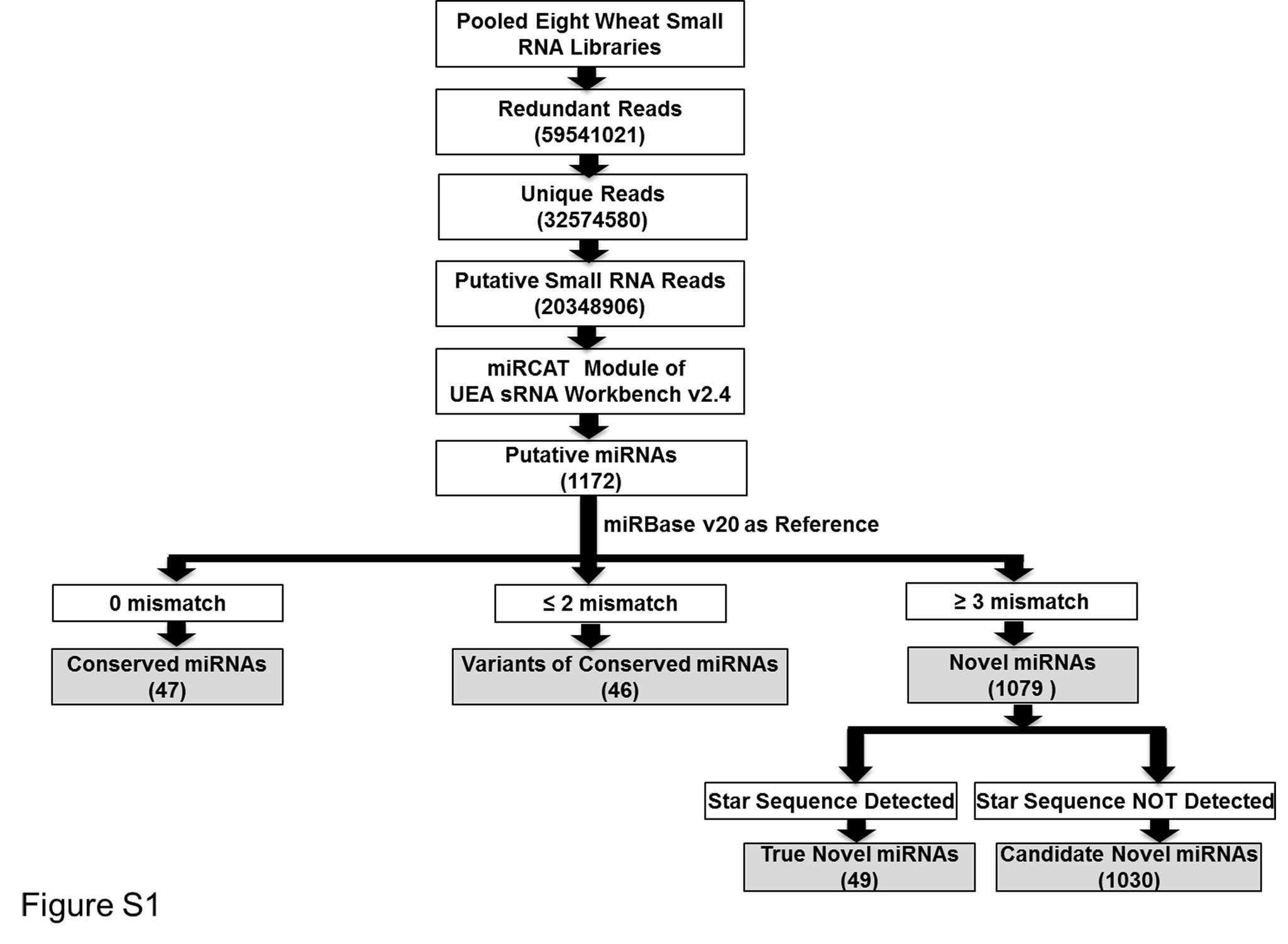

Supplement: Figure S1 — Schematic workflow for the identification of wheat miRNAs in high throughput sequence reads obtained with eight pooled wheat small RNA libraries. (TIF) [file pone.0095800.s001.tif]

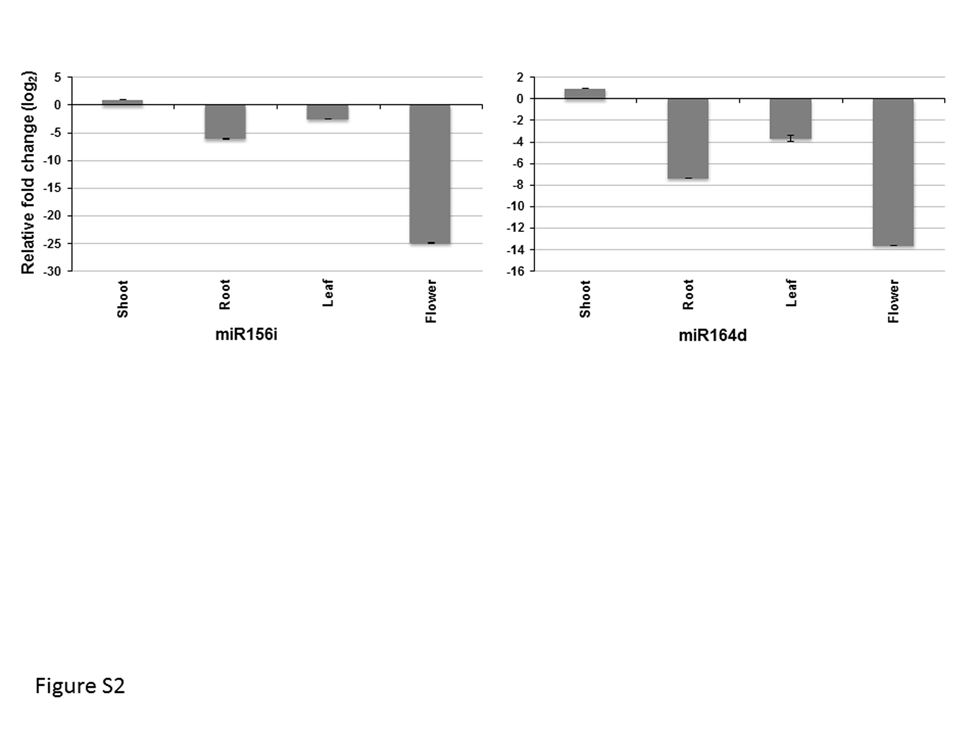

Supplement: Figure S2 — Tissue-specific expression profiling of miRNAs identified in wheat by qPCR method. PolyA tailing of total RNAs followed by cDNA synthesis and Taqman-based qPCR was employed for validation of miRNAs and normalization was carried out with wheat 5 S rRNA. Error bars represent standard error of three independent biological replicates. (TIF) [file pone.0095800.s002.tif]

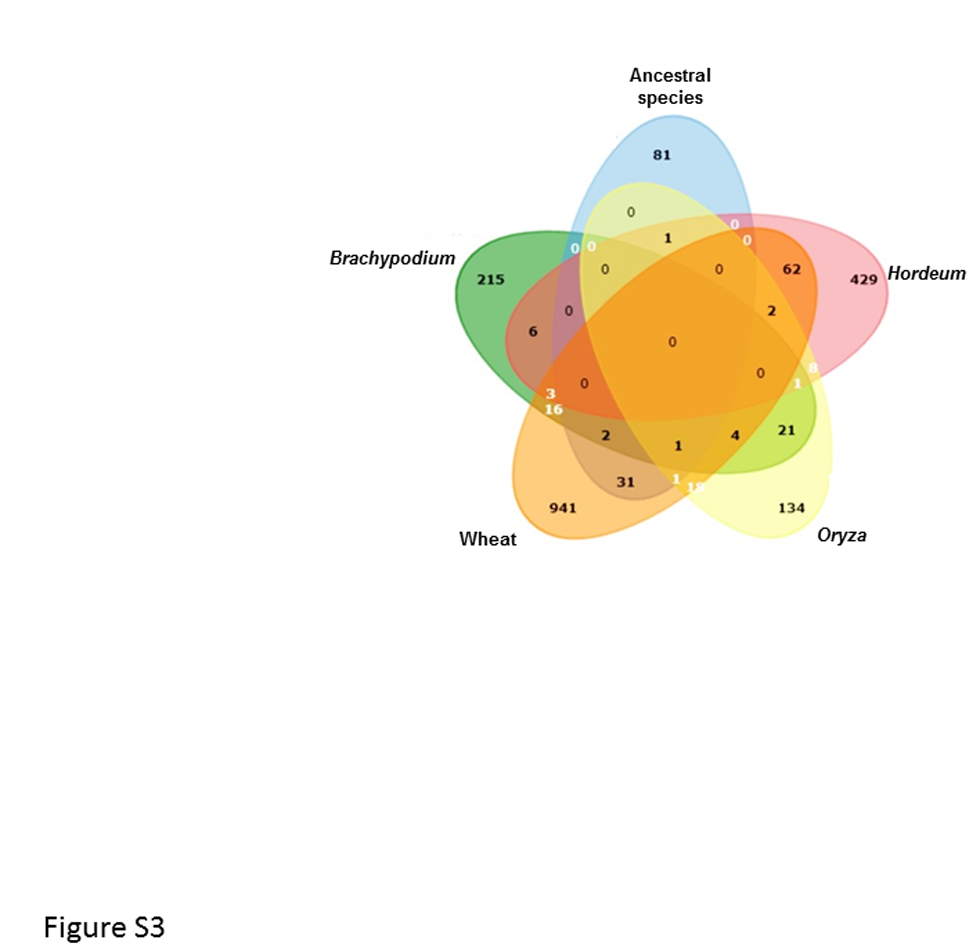

Supplement: Figure S3 — Overlap of miRNAs among wheat and related monocot species. Venn diagram representing overlap of novel miRNA population mapping onto genomic sequences of wheat, ancestral species (Triticum urartu, Aegilops speltoides and Aegilops tauschii), Brachypodium, Hordeum and Oryza. (TIF) [file pone.0095800.s003.tif]
